# Bone Morphogenetic Protein for the Healing of Tibial Fracture: A Meta-Analysis of Randomized Controlled Trials

**DOI:** 10.1371/journal.pone.0141670

**Published:** 2015-10-28

**Authors:** Jiezhi Dai, Li Li, Chaoyin Jiang, Chunyang Wang, Hua Chen, Yimin Chai

**Affiliations:** Department of Orthopedic Surgery, Shanghai Jiao Tong University Affiliated Sixth People's Hospital, Shanghai, China; Georgia Regents University, College of Dental Medicine, UNITED STATES

## Abstract

**Purpose:**

To review the evidence from RCTs on clinical outcomes and benefit of acute tibial fracture and nonunion treated with and without BMPs.

**Material:**

We searched multiple databases (MEDLINE, EMABSE, BIOSIS and Cochrane central) as well as reference lists of articles and contacted authors. Evaluated outcomes included union rate, revision rate, hardware failure and infection. The weighted and standard mean difference (WMD and SMD) or the relative risk (RR) was calculated for continuous or dichotomous data respectively. The quality of the trial was assessed, and meta-analyses were performed with the Cochrane Collaboration’s REVMAN 5.0 software.

**Results:**

Eight RCTs involving 1113 patients were included. For acute tibial fracture, BMP group was associated with a higher rate of union (RR, 1.16; 95% CI, 1.04 to 1.30) and a lower rate of revision (RR, 0.68; 95% CI, 0.54 to 0.85) compared with control group. No significant differences were found in rate of hardware failure and infection. The pooled RR for achieving union for tibial fracture nonunion was 0.98 (95% CI, 0.86 to 1.13). There was no significant difference between the two groups in the rate of revision (RR, 0.48; 95% CI, 0.13 to 1.85) and infection (RR, 0.61; 95% CI, 0.37 to 1.02).

**Conclusion:**

Study on acute tibial fractures suggests that BMP is more effective that controls, for bone union and for decreasing the rate of surgical revision to achieve union. For the treatment of tibial fracture nonunion, BMP leads to similar results to as autogenous bone grafting. Finally, well-designed RCTs of BMP for tibial fracture treatment are also needed.

## Introduction

Fractures of the tibial are mostly caused by high-energy trauma, such as motor vehicle accidents[1]. They can be either open or closed, and are associated with high prevalence of delayed union and nonunion. The management of acute tibial fractures and delayed or nonunion remains challenging by high rates of treatment failure and significant patient disability and dissatisfaction[2]. In the United States the total cost for non-union management is about 14.6 million dollars per year[3], with patients typically submitting to frequent hospital admissions and a number of interventions.

Traditional treatments of fracture fixation such as intramedullary nails and plate have been combined with biologically active substances such as autograft or bone substitutes in an effort to improve healing potential both for acute fractures and nonunions. Bone morphogenetic proteins (BMPs), discovered by Urist et al. in 1965[4], are members of the transforming growth factor beta (TGF-beta) super-family and playing a critical role in bone formation and repair. In vivo studies, multiple BMPs are expressed in fracture healing[5]. In acute fractures, BMPs act to accelerate bone union and reduce the rate of revision, while in nonunions, they are used to stimulate healing where it has not previously been successful[6].

Previous systematic reviews and meta-analyses on this problem have been discussed. According to the two meta-analyses of Wei PD[7] and Wei S[8], BMP has certain advantages in treating open tibial fractures. However, both were limited by small sample sizes with poor quality. Garrison[9] suggested that BMP may be more effective than controls for acute tibial fracture healing, however, the use of BMP for treating nonunion remains unclear. The included studies involved patients with varying diagnoses, including acute tibial fractures, tibial nonunions, critically sized defects and radial malunion. Hagen[10] discussed BMP, CaP cements and bone marrow-based bone substitute materials for the treatment of traumatic fractures of the extremities. Only three studies using BMP for fracture healing were eligible for analysis and it concluded BMP-2 was a viable alternative for treatment of open fractures. Another systematic review by Garrison 2007[11] studied clinical effectiveness and cost-effectiveness of BMPs in the non-healing of fractures and spinal fusion. Tibial fractures, scaphoid non-union and spinal fusion were reviewed and all trials were completed before 2007. Despite the high relevance of the topic, a systematic review of BMPs for the healing of tibial fracture on the basis of high quality studies is still lacking. Besides evidence for the use of BMPs in other clinical situation, the incremental effectiveness of BMPs for specific tibial fracture treatment is worth considering and a strong analysis is required using large, randomized trials to overcome these limitations.

To assess the incremental effectiveness of BMPs on fracture healing in acute fractures and nonunions compared with standards of care, we have performed this study. Furthermore, we also attempted to illuminate the limitations of current studies and to provide suggestions for further studies to evaluate these therapeutic options for the treatment of tibial fractures with BMPs.

## Materials and Methods

### Study selection

The electronic databases MEDLINE, EMABSE, BIOSIS and Cochrane central, last updated on January 31, 2014, were searched. We also searched Google Scholar and reference lists of articles. No language restrictions were applied. The search terms with BMP and tibial were retrieved in the titles, abstracts, and Medical Subject Headings. Trials were independently assessed for inclusion by three reviewers (D.J.Z., L.L., and J.C.Y.,). In cases of disagreement, a senior reviewer (C.Y.M) was consulted and a decision was made by discussion.

### Inclusion and exclusion criteria

Inclusion criteria included (1) Randomized controlled trials (RCTs) comparing use of BMP for the healing of tibial fracture with one or more current standard treatments; (2) skeletally mature patients, aged 18 and older with tibial fractures, either acute or nonunion; (3) patient treated with BMP versus surgery, BMP versus surgery with or without autograft, or BMP and bone substitutes versus surgery and bone substitutes; (4) outcomes including union rate, revision rate, hardware failure and infection; (5) >10 patients in each group; and (6) at least six-months follow-up. Exclusion criteria included (1) retrospective or nonrandomized control trials; (2) animal models and children.

### Data extraction

Data on the outcomes listed above were extracted by two reviewers (D.J.Z. and L.L.). Differences were resolved by discussion. Effective data included trial methods, populations, interventions and outcomes. Where necessary, detail was sought from the authors of the primary studies.

### Statistical analysis

The data from trials were pooled together and analyzed using the Cochrane Collaboration’s REVAMAN 5.0 software. For each study, relative risks (RR) and 95% confidence intervals (CIs) were calculated for dichotomous outcomes, and weighted mean differences (WMD) and 95% confidence intervals (CIs) were calculated for continuous outcomes. Heterogeneity of effect size across trials was tested by using I^2^ statistic. Because the test for heterogeneity had low statistical power, the presence of heterogeneity was assumed a priori, and the random effects model was used in all the analyses.

### Assessment of methodological quality and publication bias

The methodological quality of these trials was evaluated with the Cochrane Collaboration’s tool. Assessments of five main fields included sequence generation, allocation concealment, blinding, incomplete outcome data and selective outcome reporting. It was judged by answering a question, with “yes” indicating low risk of bias, “no” indicating high risk of bias, and “unclear” indicating unclear or unknown risk of bias.

We planned to draw funnel plots of primary outcomes to assess the potential publication bias. However, the small number of included studies precluded this form of analysis.

## Result

**Fig 1:**
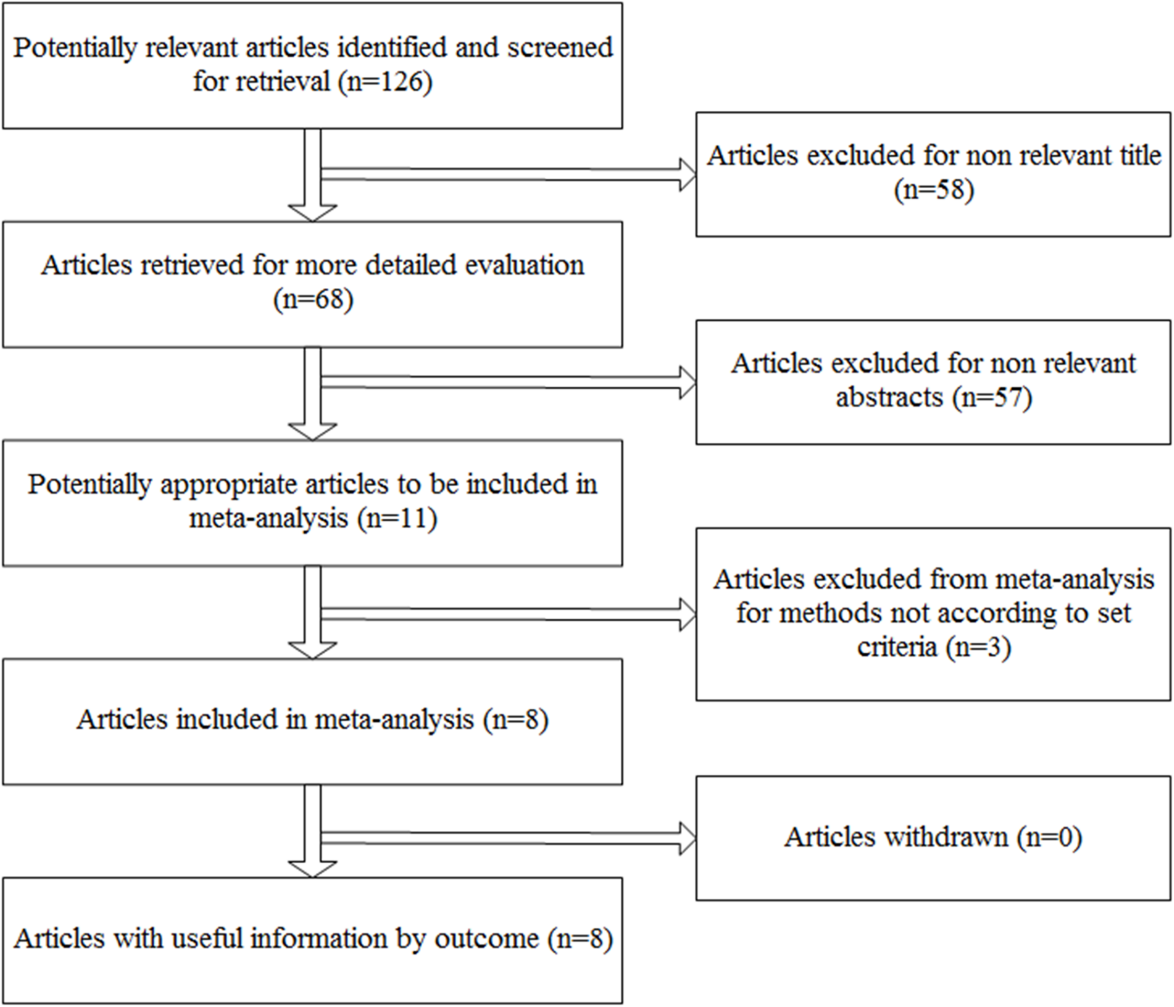
Flow chart.

126 articles were identified with use of our search strategy and the process of study selection was shown in Fig 1. After we evaluated all retrieved titles and abstracts, 11 studies remained. We excluded several studies because of the case-control or cross-sectional design or a lack of data, resulting in a total of 8 studies being included in the meta-analysis.

Details on the eight randomized, controlled trials that were included in the review are documented in Tables 1 and 2. A total of 1113 patients were included. Four trials involved 868 patients with acute tibial fractures, of which three trials include patients with open tibial fractures [12–14], and one with both open and closed tibial fracture [15]. Four trials included 245 patients with tibial fracture nonunion [16–19]. All trials were written in English, except Chen 2000 (in Chinese).

**Table 1:** Characteristics of the included trials.

Studies	Fracture type	Intervention		No. of patients		Age		Sex ratio (M %)		Follow-up	Drop-out
		BMP	Control	BMP	Control	BMP	Control	BMP	Control		
McKee 2002	Acute/Open	BMP-7	SC	62	62	NR	NR	NR	NR	6m	0
Govender 2002	Acute/Open	BMP-2: 0.75mg/mL	SC	145	147	37	37	79%	79%	12m	3.6%
		BMP-2: 1.5mg/mL		145		33		85%			
Jones 2006	Acute/Open and close	BMP-2: 1.5mg/mL	IM+ Autograft	15	15	36	38	93%	87%	12m	20%
Aro 2011	Acute/Open	BMP-2: 1.5mg/mL	SC	139	138	39.5	37.5	81%	80%	12m	16.2%
Perry 1997	Nonunion	BMP-7	IM+ Autograft	20	21	NR	NR	NR	NR	12m	0
Cook 1999	Nonunion	BMP-7	IM+ Autograft	14	16	NR	NR	77%	77%	9m	0
Chen 2000	Nonunion	BMP+NNB	IM+ Autograft	20	30	35	35	72%	72%	19m	0
Friedlaender 2001	Nonunion	BMP-7: 3.5mg	IM+ Autograft	63	61	38	34	67%	77%	24m	0

BMP: Bone morphogenetic protein; SC: Intramedullary nail fixation and routine soft-tissue management; IM: Intramedullary nail fixation; NNB: Non-organic bone; NR: Not reported.

**Table 2:** Clinical outcomes of the included trials.

Studies		Union		Revision		Hardware failure		Infection	
		BMP	Control	BMP	Control	BMP	Control	BMP	Control
McKee 2002		54	45	8	17	NR	NR	NR	NR
Govender 2002	BMP: 0.75mg/mL	75	66	51	66	25	32	31	39
	BMP: 1.5mg/mL	92		37		16		30	
Jones 2006		13	10	2	5	0	2	3	1
Aro 2011		95	92	16	17	24	21	27	15
Perry 1997		19	17	NR	NR	NR	NR	NR	NR
Cook 1999		12	15	NR	NR	NR	NR	0	1
Chen 2000		20	30	NR	NR	NR	NR	NR	NR
Friedlaender 2001		39	45	3	6	NR	NR	16	25

BMP: Bone morphogenetic protein; NR: Not reported.

Four trials [14, 17–19] used BMP-7. Three trials[12, 13, 15] used BMP-2 and one[16] with BMP and natural non-organic bone (NNB). Aro 2011 and Jones 2006 reported the treatment with a 1.5 mg/ml dose of BMP-2 (total dose of 12 mg), while Govender 2002 used BMP-2 with dose of 1.5 mg/ml (total dose of 12 mg) or 0.75 mg/ml (total dose of 6 mg). Friedlaender 2001 reported a 3.5mg dose of BMP-7 in a type 1 collagen carrier. Five trials [15–19] compared treatment with BMP with autograft and three trials [12–14] compared BMP with SC treatment (intramedullary nail fixation and routine soft-tissue management).

Methodological quality of the included trials was detailed in Table 3.

**Table 3:** Assessments of risk of bias of the randomized, controlled trials.

Studies	Sequence generation	Allocation concealment	Blinding	Incomplete outcome data	Selective outcome reporting
McKee 2002	Unclear	Unclear	Unclear	Yes	Yes
Govender 2002	Yes	Yes	Yes	Yes	Yes
Jones 2006	Unclear	Unclear	Yes	Unclear	Yes
Aro 2011	Yes	Yes	Yes	Unclear	Yes
Perry 1997	Unclear	Unclear	Unclear	Yes	Yes
Cook 1999	Unclear	Unclear	Unclear	Yes	Yes
Chen 2000	No	No	No	Yes	Yes
Friedlaender 2001	Unclear	Unclear	Yes	Yes	Yes

All trials reported a definition of successful fracture union and the union rate. Secondary outcomes included rate of surgical revision, infection and hardware failure. RCTs were grouped as either acute or nonunion of tibial fracture for meta-analysis.

### Treatment with acute tibial fractures

**Fig 2:**
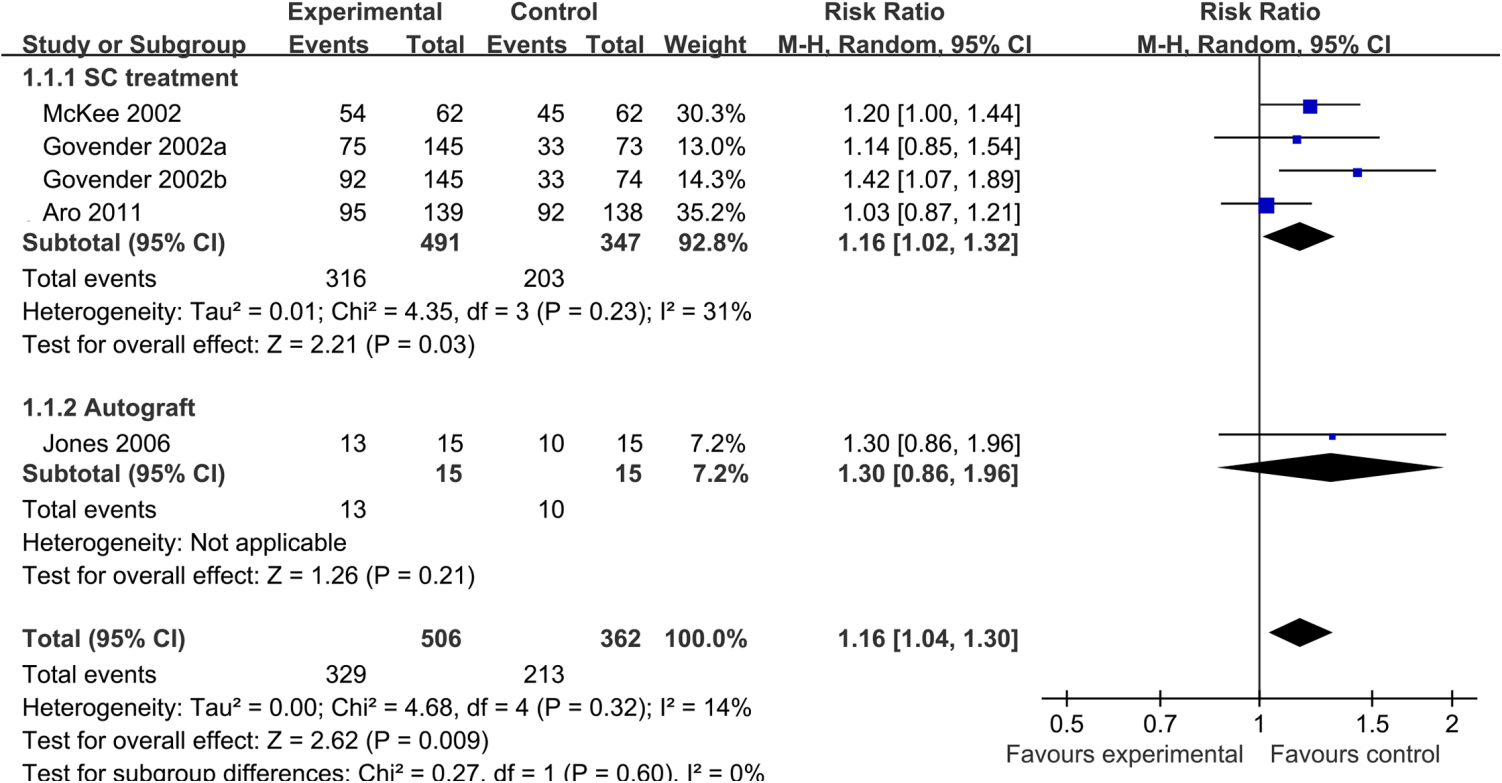
Forest plot of union rate of acute tibial fracture treated with BMP versus control group. Note: a, b: The dose of 0.75 mg/ml and 1.5 mg/ml of BMP-2 are each compared to half of the control group; SC: Intramedullary nail fixation and routine soft-tissue management.

For acute tibial fracture, four trials reported the outcome of bone union. The I^2^ statistic was 14% and the 95% confidence interval ranged from 0% (no heterogeneity) to 82% (high heterogeneity). The risk ratio for attaining union was 1.16 (95% CI 1.04 to 1.30) with applying the random-effects model. Results gave a pooled rate of 65.0% (329 of 506) in the BMP group and of 58.8% (213 of 362) in the control group. In this study, the dose of 0.75 mg/ml and 1.5 mg/ml of BMP-2 were each compared to half of the control group in the Govender 2002. Apart from Jones 2006 treated with autograft, a subgroup analysis compared BMP with SC treatment. The pooled rate of union was 64.4% (316 of 491) in BMP group and 58.5% (203 of 347) in SC group (RR, 1.16; 95% CI, 1.02 to 1.32). Similar results showed the union rate in the BMP group was significantly higher than in the SC/autograft group for acute tibial fracture (Fig 2).

**Fig 3:**
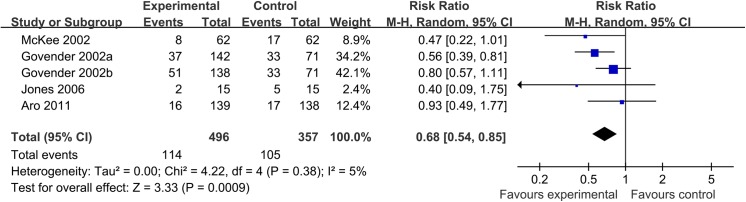
Forest plot of revision rate of acute tibial fracture treated with BMP versus control group. Note: a, b: The dose of 0.75 mg/ml and 1.5 mg/ml of BMP-2 are each compared to half of the control group.

Patients underwent surgical revision according to four trials was reported. The I^2^ statistic was 5% and the 95% confidence interval ranged from 0% (no heterogeneity) to 80% (high heterogeneity). Pooled results showed that the rate in the control group was significantly higher than in the BMP group (23.0% vs. 29.4%, respectively; RR, 0.68; 95% CI, 0.54 to 0.85) (Fig 3).

**Fig 4:**
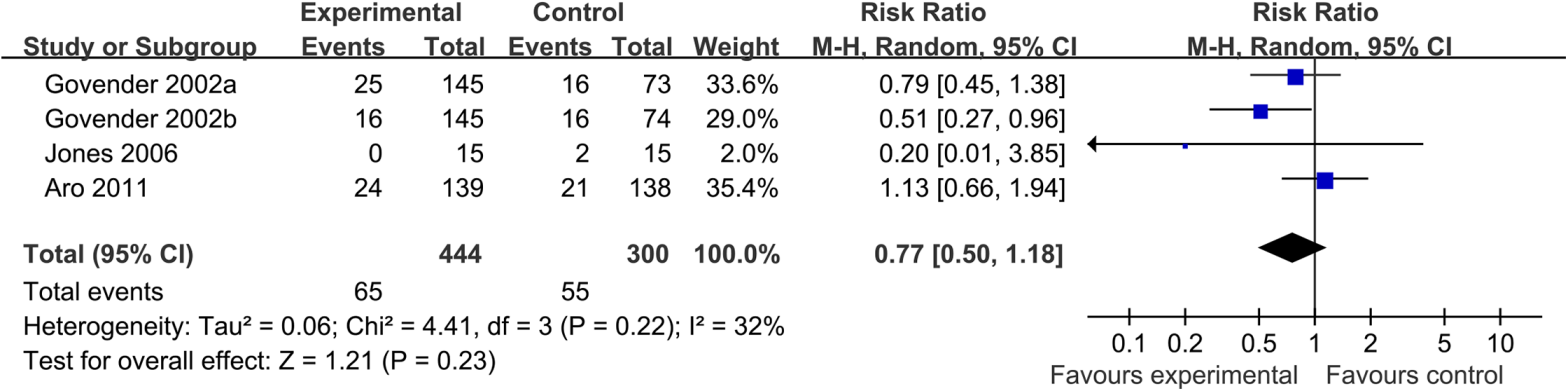
Forest plot of hardware failure rate of acute tibial fracture treated with BMP versus control group. Note: a, b: The dose of 0.75 mg/ml and 1.5 mg/ml of BMP-2 are each compared to half of the control group.

Three trials reported patients developing hardware failures. The I^2^ statistic was 32% and the 95% confidence interval ranged from 0% (no heterogeneity) to 76% (high heterogeneity). There was no significant difference in the BMP groups between the control group (RR, 0.77; 95% CI, 0.50 to 1.18) (Fig 4), but a trend favoring control group was observed.

**Fig 5:**
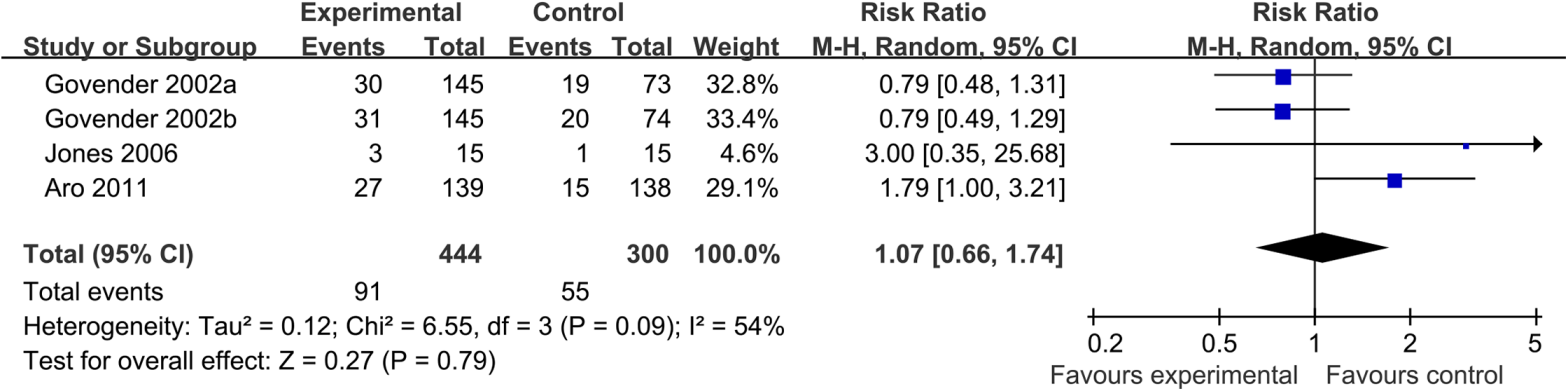
Forest plot of infection rate of acute tibial fracture treated with BMP versus control group. Note: a, b: The dose of 0.75 mg/ml and 1.5 mg/ml of BMP-2 are each compared to half of the control group

Infection-risk analysis across three trials showed no significant differences were found when comparing BMP groups with control groups (20.5% vs. 18.3%, respectively; RR, 1.07; 95% CI, 0.66 to 1.74) (Fig 5). The I^2^ statistic was 54% and the 95% confidence interval ranged from 0% (no heterogeneity) to 85% (high heterogeneity).

### Treatment with tibial fracture nonunion

**Fig 6:**
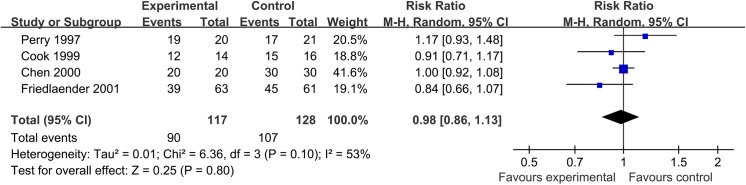
Forest plot of union rate of tibial nonunion treated with BMP versus control group.

For nonunion to tibial fracture, three trials were included in the analysis. There was a significant heterogeneity (I^2^ = 53%) between trials and the 95% confidence interval ranged from 0% (no heterogeneity) to 84% (high heterogeneity). This heterogeneity could lower when removing the trial Friedlaender 2001. The pooled RR for achieving union was 0.98 (95% CI, 0.86 to 1.13) (Fig 6). The pooled data demonstrated no significant difference was found when comparing BMP with autograft for nonunion fracture treatment.

**Fig 7:**
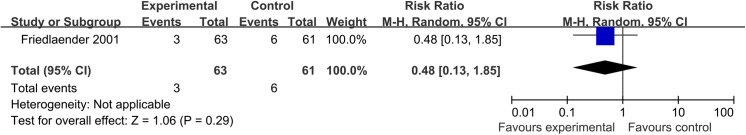
Forest plot of revision rate of tibial nonunion treated with BMP versus control group.

**Fig 8:**
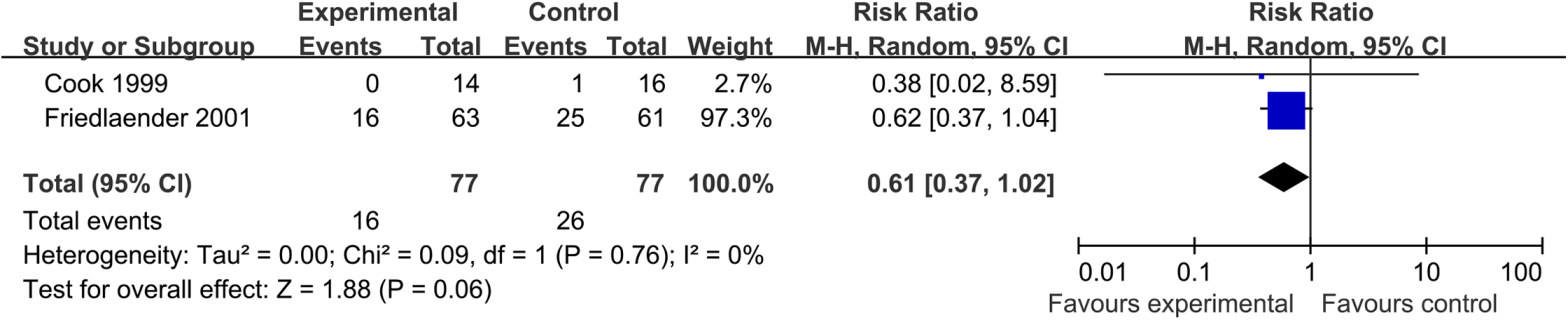
Forest plot of infection rate of tibial nonunion treated with BMP versus control group.

Only one trial in the nonunion group reported the data on surgical revision and two trials reported the rate of infection. The pooled rate of surgical revision was 4.8% (3/63) in the BMP group and 9.8% (6/61) in the control group (RR, 0.48; 95% CI, 0.13 to 1.85). Two trials found no significant difference between the two groups in the rate of infection (RR, 0.61; 95% CI, 0.37 to 1.02); however, most of this evidence was dominated by the reuslt of Friedlaender 2001. The results of these analyses were provided in Figs 7 and 8.

## Discussion

Eight randomized controlled trials involving the treatment of tibial fractures fulfilled the inclusion criteria for this meta-analysis. Four trials involved patients with acute tibial fracture, and there was some evidence for improved union rate, without requiring a surgical revision, of BMP comparing with SC/autograft treatment. Four trials included patients with tibial fracture nonunion. No evidence of benefit was found when BMP comparing with bone grafts, for attaining union. The data also showed the BMP group was significantly associated with a lower risk of surgical revision for the acute tibial fracture, while no significant difference was seen in hardware failure and infection rate between groups. For the treatment of tibial fracture nonunion, as a small number of patients involved, no significant difference was found on the rate of surgical revision and infection.

In acute tibial fracture, delayed or nonunion, it was possible to induce bone at the fracture site to support healing [9]. This specialized process normally regenerated bone in a well-orchestrated biological process that regained skeletal integrity. Autograft from the iliac crest was considered the current gold standard for bone repair and reconstruction [20] with both histo-compatible and non-immunogenic. However, harvesting the grafts was associated with donor-site morbidity, particularly chronic pain and dissatisfaction with appearance[21]. BMP was soluble bone matrix glycoprotein that stimulated the steps from stem cell differentiation to osteoblastic mature cells. It seemed to play an important role in the healing process and act as autogenous bone graft substitutes. Flierl et al.[22] doubted the indication spectrum of BMP with controversy, particularly regarding its questionable safety and efficiency profile.

Previous clinical studies assessing BMP in fracture and nonunion management were limited. Westerhuis et al.[23] reviewed a larger number of non-randomized studies using different BMPs. Although the results were impressive for bone healing, they were largely anecdotal and generally lack control groups. In 2002, the results of the BESTT multicenter prospective randomized trial showed a significant decrease in surgical revision and a higher union rate in patients with BMP-2 treatment for acute open tibial fracture[13]. Again, as reaming was likely to producing bone ‘dust’, which was a form of bone graft and help healing, this should have been considered in this study protocol.

In 2012, a meta-analysis by Wei et al.[8] reported the use of rhBMP-2 in open tibial shaft fractures. The authors suggested that rhBMP-2 added to intramedullary nail fixation of open tibial fractures could reduce the rate of surgical revisions and total health care costs. Their meta-analysis included four trials, two of which were omitted from the present meta-analysis because of inappropriate data (the data of Swiontkowski was combined and analyzed from Govender 2002 and US study group. Communication from Prof Swiontkowski confirmed that this trial was sponsored by Wyeth and has never been published independently as it was underpowered for the pre-determined endpoints) [24], and insufficient reporting of results (an economic evaluation study and the clinical data collected from Govender 2002) [25]. We included six extra trials that did fulfill our strict inclusion criteria and performed a new meta-analysis to review the clinical data currently available on the use of BMPs in both acute tibial fractures and nonunion. Nevertheless, the results reported by Wei et al.[8] were similar to ours; besides, BMP remained as effective as autograft for nonunion fracture treatment, with similar union rate (RR = 0.98).

Our meta-analysis found no significant difference in union rate for BMP, compared with autograft, for tibial fracture nonunion. It was also confirmed in other studies. In tibial fracture nonunion, similar and even higher success rates were attained by Calori et al.[26] in 2008, Zimmermann et al. [3] in 2009 and others. It concluded that BMPs may provide a viable alternative to autogenous bone grafting for the treatment of long bone nonunion [27]. There was, however, heterogeneity between these trials. It decreased from 53% to 24% when removing the trial Friedlaender 2001. It was probably due to selection bias when a study group contained greater numbers of nonunion than the control group. This was the case for the largest trial in this group.

In the BESTT study [13], it was inadequately powered to demonstrate a between-group difference in the reamed-nailing subpopulation. A more recent RCT, with the similar design to the BESTT, failed to demonstrate improved fracture-healing when used with reamed-nailing and rhBMP-2 for the treatment of tibial fractures[12]. It suggested that larger randomized trials that involved reamed-nailing and BMPs may confirm the differences observed.

Only one trial Govender 2002 compared two dose of BMP-2 and tried to find the effects with dose-dependent. Subsequent studies followed the results with the use of 1.5mg/mL dose. In our meta-analysis, the dose of 0.75 mg/ml and 1.5 mg/ml of BMP-2 were each compared to half of the control group. Although, the results for the higher dose of 1.5mg/mL were more impressive than those for the 0.75mg/mL dose, an interactive test did not find any statistically significant difference. Luginbuehl et al.[28] reported a clear species-specific dose response using rhBMP-2 ranging from 25ug/mL in rodents to 50ug/mL in dogs, 100ug/mL in non-human primates and 800ug/mL in humans. The doses currently used were supra-physiological and we wondered the long-term effects of this would be. One explanation was that supra-physiologic concentrations might be required to overcome the effects of natural inhibitors of growth factors[29]. This should be considered for dose stratification in the future study.

No clear answer was found in the study which of the BMPs was the optimal candidate to help bone healing in acute tibial fractures, or nonunion. Vaibhav et al.[30] concluded using BMP-7 in cases of tibial non-union and BMP-2 in cases of acute tibial fractures. Among the included RCTs, McKee 2002 compared open tibial fractures treated with either standard closure or BMP-7. The results suggested that rhBMP-7 to acute open tibial fractures was technically feasible and not associated with any increase in adverse events[14]. Given that only BMP-2 and BMP-7 were approved for clinical application currently, further investigation with more specific BMP subtypes were required.

## Conclusion

Study on acute tibial fractures suggests that BMP is more effective that controls, for bone union and for decreasing the rate of surgical revision to achieve union. For the treatment of tibial fracture nonunion, BMP leads to similar results to as autogenous bone grafting. It indicates that BMP is not the ultimate solution in the challenging field of acute tibial fracture and non-union treatment. In other words, the use of BMPs has supplied a biological component to the treatment options available[31]. Finally, well-designed RCTs of BMP for tibial fracture treatment are also needed.

## Supporting Information

S1 PRISMA Checklist (PDF)

S1 File Electronic Supplementary. (DOC)
