# Supplementary material for: Bone Morphogenetic Protein for the Healing of Tibial Fracture: A Meta-Analysis of Randomized Controlled Trials
Source: PLoS One. 2015 Oct 28;10(10):e0141670. doi: 10.1371/journal.pone.0141670 (PMC4624800; doi:10.1371/journal.pone.0141670)
Supplement: S1 File — (DOC) [file pone.0141670.s002.doc]

Electronic Supplementary Material Table: the exclusion of 3 studies

| First author | Study | Reason of exclusion |
| --- | --- | --- |
| Alt 2009 | A health economic analysis of the use of rhBMP-2 in Gustilo-Anderson grade III open tibial fractures for the UK, Germany, and France. Injury 40: 1269-1275. | Lack of data |
| Swiontkowski 2006 | Recombinant human bone morphogenetic protein-2 in open tibial fractures. A subgroup analysis of data combined from two prospective randomized studies. J Bone Joint Surg Am 88: 1258-1265. | Lack of data |
| Zimmermann 2007 | Therapeutic outcome in tibial pseudarthrosis: bone morphogenetic protein 7 (BMP-7) versus autologous bone grafting for tibial fractures. [Unfallchirurg.](http://www.ncbi.nlm.nih.gov/pubmed/17989951) 2007 Nov;110(11):931-8. | Case-control or cross-sectional design |
